# Volumetric Absorptive Microsampling (VAMS) for Targeted LC-MS/MS Determination of Tryptophan-Related Biomarkers

**DOI:** 10.3390/molecules27175652

**Published:** 2022-09-01

**Authors:** Michele Protti, Marco Cirrincione, Roberto Mandrioli, James Rudge, Luca Regazzoni, Valeria Valsecchi, Claudia Volpi, Laura Mercolini

**Affiliations:** 1 Research Group of Pharmaco-Toxicological Analysis (PTA Lab), Department of Pharmacy and Biotechnology (FaBiT), Alma Mater Studiorum–University of Bologna, Via Belmeloro 6, 40126 Bologna, Italy; 2 Department for Life Quality Studies (QuVi), Rimini Campus, Alma Mater Studiorum–University of Bologna, Corso d’Augusto 237, 47921 Rimini, Italy; 3 Neoteryx LLC, 421 Amapola Ave, Torrance, CA 90501, USA; 4 Department of Pharmaceutical Sciences, University of Milan, Via Mangiagalli 25, 20133 Milan, Italy; 5 Department of Neuroscience and Reproductive and Odontostomatological Sciences, University of Naples Federico II, Via Pansini 5, 80131 Naples, Italy; 6 Department of Medicine and Surgery, University of Perugia, Piazzale Gambuli 1, 06132 Perugia, Italy

**Keywords:** volumetric absorptive microsampling (VAMS), LC-MS/MS, tryptophan catabolism, biomarkers, neurodegenerative diseases

## Abstract

L-Tryptophan (TRP) metabolites and related biomarkers play crucial roles in physiological functions, and their imbalances are implicated in central nervous system pathologies and neurodegenerative diseases such as amyotrophic lateral sclerosis (ALS), Alzheimer’s disease, Parkinson’s disease, schizophrenia and depression. The measurement of TRP metabolites and related biomarkers possesses great potential to elucidate the disease mechanisms, aid preclinical drug development, highlight potential therapeutic targets and evaluate the outcomes of therapeutic interventions. An effective, straightforward, sensitive and selective liquid chromatography-tandem mass spectrometry (LC-MS/MS) method was developed for the simultaneous determination of 24 TRP-related compounds in miniaturised murine whole blood samples. Sampling and sample pretreatment miniaturisation were achieved thanks to the development of a volumetric dried blood microsampling approach. Volumetric absorptive microsampling (VAMS) allows the accurate sampling of microvolumes of blood with advantages including, but not limited to, minimal sampling invasiveness, logistical improvements, method sustainability in terms of solvents and energy consumption, and improvement of animal studies in the framework of the 3Rs (Replacement, Reduction and Refinement) principles on animal welfare. The VAMS-LC-MS/MS method exhibited good selectivity, and correlation coefficient values for the calibration curves of each analyte were >0.9987. The limits of quantitation ranged from 0.1 to 25 ng/mL. The intra- and inter-day precisions in terms of RSD were <9.6%. All analytes were stable in whole blood VAMS samples stored at room temperature for at least 30 days with analyte losses < 14%. The developed method was successfully applied to the analysis of biological samples from mice, leading to the unambiguous determination of all the considered target analytes. This method can therefore be applied to analyse TRP metabolites and related biomarkers levels to monitor disease states, perform mechanistic studies and investigate the outcomes of therapeutic interventions.

## 1. Introduction

**Figure 1:**
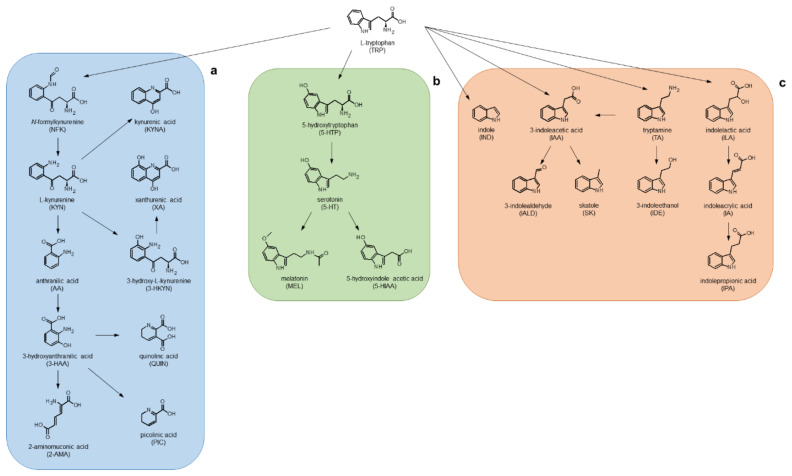
Schematic illustration of TRP metabolism through the (**a**) KYN and (**b**) 5-HT pathways, and (**c**) gut TRP catabolism.

In addition to protein synthesis, most of the L-tryptophan (TRP) in mammals is metabolised along the kynurenine (KYN) and indole (IND) pathways. Within the former pathway, more than 95% of TRP is oxidised to *N*-formylkynurenine (NFK) by tryptophan-2,3-dioxygenase or indoleamine-2,3-dioxygenase (IDO), followed by the synthesis of the first stable intermediate KYN. Subsequently, KYN is degraded to kynurenic acid (KYNA), 3-hydroxykynurenine (3-HKYN) and anthranilic acid (ATA) by kynurenine aminotransferase (KAT), kynurenine 3-monooxygenase and kynureninase, respectively (Figure 1a). The KYN pathway has been implicated in the pathophysiology of several diseases including neurodegenerative disorders [1], cancer, infection [2], autoimmune disorders [3] and psychiatric disorders [4]. Moreover, TRP is also the obligatory substrate for the production of several important bioactive neurotransmitters and neurotransmitter-like compounds. For example, TRP is a substrate for the synthesis of serotonin (5-hydroxytryptamine, 5-HT) in the brain and gut through the 5-HT pathway, and of melatonin (MEL) in the pineal gland (Figure 1b) [5]. Finally, the cross-talk between the host and the gut microbiota has been widely studied in recent decades (Figure 1c). Gut-derived small molecules, such as TRP-related IND and its derivatives, interact with the host and exert a variety of local and heterotopic biological effects by circulating in the bloodstream [6].

The rather broad spectrum of biological activities and potential implications of TRP-related compounds on a wide array of pathologies and conditions underlines the importance of the targeted assessment of changes in the levels of TRP metabolites and related neurotransmitters for research purposes in several fields. The prerequisites are the simultaneous analysis of compounds with sufficient sensitivity and specificity, and the feasibility of sampling and pre-analytical steps. Furthermore, an acceptable analytical run-time is necessary to allow sufficient sample throughput, thereby fulfilling research needs. For measurement standardisation and reproducibility, sample preparation procedures should be standardised as well, and made as feasible as possible.

To overcome the inherent disadvantages associated with classic fluid whole blood analysis, several works have exploited microsampling approaches, such as dried blood spot (DBS), originally developed for neonatal screening for inborn errors of metabolism [7]. DBS involves a minimally invasive collection of blood (less than 50 µL), usually obtained from a heel- or finger-prick, spotted onto dedicated cards, and followed by simplified storage and transportation procedures. These samples, simply dried at room temperature, usually do not require cryopreservation and are quite stable over time due to the absence of water. After minimal training, DBS collection can be easily performed by non-qualified operators, thus representing a concrete, promising perspective for point-of-care or self- and home-sampling. Due to its many advantages, DBS has been applied to many fields of bioanalysis, including pharmacokinetic studies, in order to improve animal welfare and reduce the number of animals needed [8], or to therapeutic drug monitoring (TDM) to promote frequent and feasible patient sampling [9,10]. Moreover, microsampling has also been exploited for proteomics to assess protein stability [11], for lipidomic studies [12] and, more generally, for biomarker discovery [13,14]. An interesting miniaturised sampling technique is volumetric absorptive microsampling (VAMS), which holds all the advantages of DBS while providing additional ones, mostly related to sampling volume accuracy and haematocrit (HCT)-dependent volumetric bias. VAMS devices are able to sample a fixed amount of fluid (10, 20, 30 µL) by means of a hydrophilic polymer tip, thus allowing accurate blood collection directly from a capillary blood drop, regardless of its density and viscosity. VAMS is not affected by HCT-dependent issues and makes the sampling procedure very feasible [15,16,17]. Moreover, the use of VAMS in animal studies supports the principle of the three Rs (3Rs, Replacement, Reduction and Refinement) by reducing the animal sample size and minimising the potential pain, allowing serial sampling from the same animal and also reducing individual variability [18]. After drying/storage at room temperature, the VAMS device tip can undergo extraction with different solvents or solvent mixtures by means of various extraction procedures [19]. The VAMS technology has been successfully applied to several analytical challenges, such as pharmacokinetics [20], therapeutic drug monitoring (TDM) [21,22] and analysis of drugs of abuse [23,24], and to different biological fluids [25,26,27,28,29,30]. VAMS has also been applied to the discovery of protein biomarkers [31] and to the determination of amino acids and organic acids in metabolomic approaches [32]. Nevertheless, to the best of our knowledge, this innovative and promising technique has never been studied for targeted TRP-related biomarker analysis applied to murine samples.

In this study, a targeted quantitative analysis in whole blood microsamples has been carefully investigated using the VAMS technology, to simultaneously determine 24 selected TRP compounds belonging to the KYN pathway, the IND and 5-HT pathways or the gut TRP metabolism. The selected target compounds were the following: TRP, NFK, KYN, ATA, KYNA, 3-HKYN, xanthurenic acid (XA), 3-hydroxyanthranilic acid (3-HAA), quinolinic acid (QUIN), picolinic acid (PIC), 2-aminomuconic acid (2-AMA), 5-hydroxytryptophan (5-HTP), 5-HT, 5-hydroxyindole acetic acid (5-HIAA), MEL, IND, tryptamine (TA), 3-indoleethanol (IDE), 3-indolepropionic acid (IPA), indolelactic acid (ILA), 3-indoleacetic acid (IAA), skatole (SK), 3-indolealdehyde (IALD), and indoleacrylic acid (IA). VAMS samples were subjected to carefully optimised sample treatment to obtain satisfactory clean-up and extraction and coupled to an original LC-MS/MS targeted quantitative method, qualified for this purpose. The aim of this work was to evaluate, for the first time, the VAMS potential as a viable alternative strategy for whole blood microvolume sampling for targeted TRP-related biomarker assessment. As a proof-of-concept, the present method was successfully applied to the analysis of VAMS samples from mice leading to the unambiguous determination of all the considered target biomarkers.

Overall, the novelty aspect of this research work is focused on the evaluation of the use of VAMS for targeted biomarker analysis, and namely for the simultaneous LC-MS/MS analysis of 24 TRP-related metabolites of interest, with a particular focus on the optimisation of sampling, drying, storage and pretreatment protocols. The method has been evaluated in terms of absolute recovery, precision, carryover, selectivity, linearity and sensitivity. Importantly, the stability of the metabolites in dried whole blood VAMS microsamples was assessed to define an appropriate experimental setup and to evaluate actual opportunities for the application of VAMS in targeted biomarker studies.

## 2. Results and Discussion

### 2.1. Liquid Chromatography and Mass Spectrometry

MS and MS/MS spectra of the analytes and ISs were acquired in the 50–600 *m*/*z* range by direct infusion in the ESI source of 1 μg/mL solutions at 10 μL/min, using a mixture of 0.3% formic acid (FA) in acetonitrile (ACN) and 0.3% FA in water (50/50, *v*/*v*) for dilutions. All spectra were acquired using both ESI+ and ESI− ionisation modes, in order to choose the best conditions for each analyte and IS. For each analyte, the most abundant ion on the ion scan spectra was selected as the precursor ion, while for product ions, the most abundant fragment ion was used for quantitative purposes, while the second most abundant ion was exploited for identity confirmation. A complete overview of MRM transitions and MS/MS conditions is reported in Table 1. Four ISs, namely TRP-d5 (IS1), KYNA-d5 (IS2), 5-HT-d4 (IS3) and IND-d7 (IS4), were selected in order to cover the full spectrum of the target analytes from the qualitative and quantitative perspectives.

A suitable chromatographic setup was then investigated for the simultaneous analysis of the 24 TRP-related biomarkers chosen for this study. Various reverse-phase columns and mobile phase conditions were tested and it was observed that the most hydrophilic compounds could be retained at a higher degree by a pentafluorophenyl (PFP) sorbent than by C8, C18 or cyanopropyl (CN) ones. Therefore, a PFP column was selected for further analysis. The mobile phase was then optimised to improve retention, peak shape and separation. First, 0.3% FA was chosen as an acidic additive as a suitable compromise for satisfactory retention, peak shape and MS signal. In order to keep a constant FA concentration and more reproducible MS ionisation throughout a gradient elution, the same amount of FA was added to both water (component A) and ACN (component B). As a final development step, the composition gradient was finely tuned in order to allow satisfactory chromatographic peak resolution and a reasonable chromatographic run time. The optimised gradient conditions were as follows: 0–3.0 min, 2% component B, 3.0–4.5 min, linear gradient from 0% to 50% component B, 4.5–7.5 min, 50% component B, 7.5–9.0 min, from 50% to 95% component B, 9.0–12.0 min at 95%, 12.0–13.5 min from 95% to 2% component B, and 13.5–16.0 min at 2% for column re-equilibration. The flow rate was 0.25 mL/min, and the injection volume was 5 μL.

In these working conditions, TRP-related compounds produced sharp and symmetric peaks for all analytes, with a total chromatographic run of 16 min, with sufficient sensitivity and selectivity.

### 2.2. VAMS Microsample Pretreatment

Currently, there is no consensus guideline for endogenous substance assay validation [33,34]. Since the selected analytes are endogenous substances in biological matrices, it is not possible to obtain a completely analyte-free authentic matrix for method development and validation. In the present study, method development and validation were carried out exploiting the standard addition method by using blank and fortified blank blood VAMS samples obtained from healthy mice.

VAMS sampling and drying times, as well as volume accuracy for whole blood were taken for granted according to the results of previous reports [23]. Briefly, the average sampled volume was 10.3 μL and volumetric accuracy (bias) was in the −8.7–8.1% range, with precision (% CV) in the 2.8–5.9% range for all contact times longer than 3 s with no oversampling effects. As for drying time assessed by gravimetric analysis, under complete ventilation at RT or enclosed in the provided clamshell packages, the samples were dried within 0.75 and 1 h, respectively.

Careful optimisation of the extraction procedure was carried out in order to maximise the recovery of the TRP-related compounds object of this study from fortified blood VAMS samples. The type of organic solvent, the aqueous percentage and the pH were investigated. ACN and methanol (MeOH) were tested as the organic solvents in various concentrations (40–100%). Moreover, the use of acidic and basic additives (namely FA and ammonium hydroxide, 0.1–1%) was studied.

**Figure 2:**
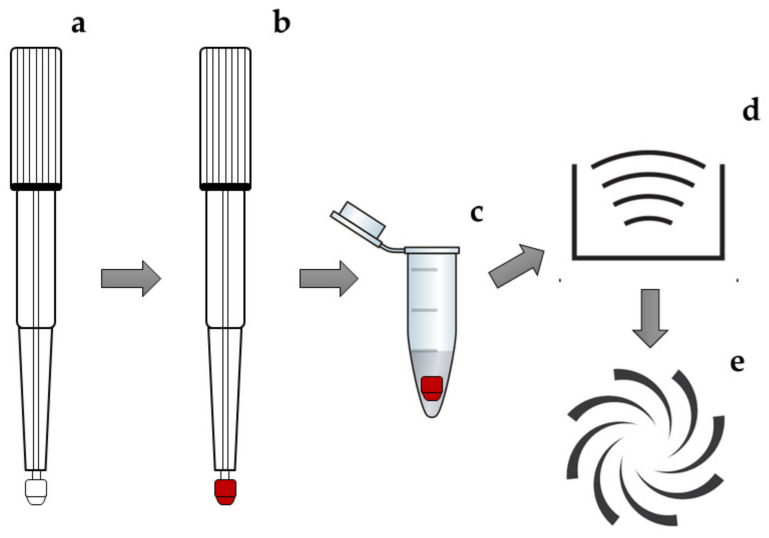
Representation of VAMS workflow: (**a**) Unused VAMS sampler, (**b**) VAMS sampler after accurate collection of 10 μL whole blood and drying at RT, (**c**) sampled VAMS tips extracted in a proper extraction media under (**d**) UAE and (**e**) VAE.

The observed signal-to-noise (S/N) ratios of the target analytes were generally higher with an extraction media containing ACN compared to the analyte signals obtained using the same amount of MeOH. Moreover, the response (in terms of S/N) of all compounds improved with the addition of an aqueous portion in the extraction mixture, reaching the best performance at a percentage of 35%. Higher percentages of water resulted in haemolysis, pink coloured extracts, a higher matrix effect and lower signal intensities. Moreover, the presence of ammonium hydroxide (in the 0.1–1% range) resulted in lower peak areas, whereas no significant effect was observed when FA (in the 0.1–1% range) was used as an additive for the extraction mixture. Regarding the extraction procedure itself, several workflows were tried: Ultrasound-assisted extraction (UAE), vortex-assisted extraction (VAE) and their combinations, applied for different times and at different potencies. The best results were obtained with a combination of UAE at 40 kHz for 10 min and VAE for 10 min. A low extraction volume (100 µL) was sufficient to obtain a satisfactory compromise between analyte extraction from blood VAMS, sample clean-up and required sensitivity (Figure 2).

### 2.3. VAMS-LC-MS/MS Method Qualification for Targeted Biomarker Assessment

#### 2.3.1. Absolute Recovery, Precision, Matrix Effect and Carryover

The absolute recovery of the TRP-related target analytes from whole blood VAMS microsamples exploiting the optimised extraction protocol was determined on blood VAMS samples fortified at three concentration levels, representative of the calibration curves for each analyte, by comparing pre-extraction and post-extraction fortified samples and expressed as a percentage. High absolute recoveries (> 85%) were obtained for all the analytes (>91% for the ISs) in combination with good reproducibility taking into account three concentrations, always obtaining RSD values lower than 9.6% (<7.3% for the ISs) (Table 2).

The standard addition method used to fortify blood VAMS samples considers matrix effects by relying on the assumption that all concentration levels (both endogenous and fortified) are subjected to a proportional ion enhancement or suppression effect because each sample (calibration and QC samples) contains the same amount of co-eluting matrix compounds. For all target analytes, the matrix effect was in the 87–96% range, as reported in Table 2. No carryover was observed for VAMS, as no signal higher than the background noise at the retention times and *m/z* of the analytes was observed when injecting a blank solvent after analysing a sample fortified with the highest concentration of the respective calibration curves (*n* = 3).

#### 2.3.2. Selectivity, Linearity and Sensitivity

The developed method was deemed to be selective since chromatographic peaks of all the target analytes were satisfactorily resolved and unambiguously identified by MRM exclusive transitions. Method sensitivity on VAMS samples was between 0.1 and 25 ng/mL in terms of the limit of quantitation (LOQ). Method linearity was satisfactory, with correlation coefficients (r^2^) always higher than 0.9987 (see Table 3). Linearity assays (carried out in triplicate for each concentration) were assessed on whole blood VAMS samples from mice, fortified by the standard addition method. Experimental LOQ (and thus linearity range) values assessed by the standard addition method were inherently proportional to the baseline analyte levels observed in the mice blood VAMS samples used for method development and qualification. As an example, the theoretical LOQ of TRP would have been way much lower (in the sub-ng/mL range), but LOQ was calculated as the lowest concentration of analyte added to the samples that can be reliably quantified with acceptable accuracy (± 20%) and precision (%RSD < 20%). In addition, stressing method sensitivity for analytes expected in mouse blood at high concentrations would have been outside the scope of the present work.

#### 2.3.3. Stability

Analyte stability in dried whole blood VAMS micromatrices is generally enhanced in comparison to fluid matrices, as reported in previous works [19]. However, in targeted biomarker assessment studies, analyte stability is of the utmost importance in order to assure that observed differences between samples are due to variations in biochemical pathways. In this study medium-term stability, as well as benchtop and autosampler stability, were tested in blood VAMS. Medium-term stability was assessed in dried micromatrices stored at RT (24 ± 4 °C). After 30 days, dried micromatrices consistently provided satisfactory stability (in the 86–95% range). In addition to the satisfactory data obtained in terms of stability, it was possible to observe a greater (albeit minimally significant in absolute terms) instability trend of acidic compounds with stability after 30 days between 87% and 91% when compared to other compounds, with stability results in the 90–94% range. In general, the results obtained highlight how the adoption of particular precautionary measures in the storage protocol of VAMS samples is not necessary for a time span of 30 days prior to analysis, thus avoiding logistical complications and increased costs due to the need to store the dried samples at controlled temperatures. Benchtop and autosampler stability at the same temperature were also good (recovery > 85%). The good stability results obtained offer promising opportunities for the application of VAMS in targeted biomarker studies. In this way, observed quali-quantitative variations among samples could be attributed with greater confidence to variations in biochemical processes without potential biases caused by variations in storage time and temperature.

### 2.4. Analysis of Real Samples and Accuracy

**Figure 3:**
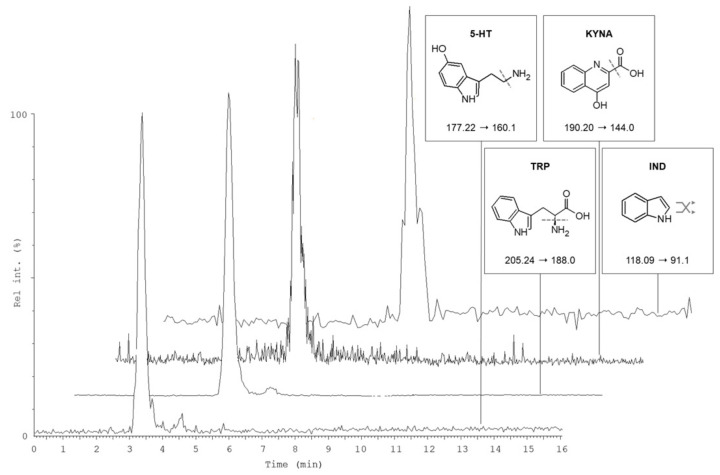
LC-MS/MS chromatogram of a whole blood VAMS sample from a mouse (female). The figure shows a selection of four representative MRM channels: 5-HT (189 ng/mL), TRP (6544 ng/mL), KYNA (9.4 ng/mL) and IND (1.2 ng/mL).

The developed VAMS-LC-MS/MS methodology was applied to the analysis of TRP and 23 TRP-related metabolites in murine whole blood microsamples. The typical LC-MS/MS chromatograms of a VAMS sample obtained from a healthy mouse not subjected to treatments, fortified with the ISs and subjected to sampling, drying, pretreatment and analysis are shown in Figure 3. Concentrations of the target analytes in murine whole blood samples from three animals (one male and two females) are reported in Table 4.

Accuracy assays were also performed on real samples, analysing additional replicates after spiking with different analyte concentrations. Very good accuracy was obtained, with absolute recovery values always in the 90–107% range. To the best of our knowledge, there are no reported data on blood concentrations of TRP-related biomarkers assessed by VAMS microsampling coupled to LC-MS/MS. Using this feasible, yet reliable microsampling-based analytical method, TRP metabolites belonging to the KYN pathway, the IND and 5-HT pathways or the gut TRP metabolism were determined with proper sensitivity and good accuracy.

## 3. Materials and Methods

### 3.1. Chemicals and Standard Solutions

TRP, NFK, ATA, KYNA, 3-HKYN, XA, 3-HAA, QUIN, PIC, 5-HTP, 5-HT, 5-HIAA, MEL, IND, TA, IDE, IPA, ILA, IAA, SK, IALD, and IA, as well as internal standards (namely TRP-d5—IS1, KYNA-d5—IS2, 5-HT-d4—IS3, IND-d7—IS4), pure powders (all >99% purity), ACN, MeOH, FA, ammonium hydroxide (all reagents for mass spectrometry) and other solvents used for sample preparation (all analytical grade) were purchased from Merck Life Science (Milan, Italy), while 2-AMA was synthesised in-house.

Ultrapure water (18.2 MΩ∙cm) was obtained by means of a Milli-Q apparatus from Merck Millipore (Darmstadt, Germany). Analyte and IS stock solutions (1 mg/mL) were prepared by dissolving suitable amounts of pure powders in MeOH and kept at −20 °C when not in use; the corresponding standard solutions were prepared daily by dilution with a mixture of water and ACN (50:50, *v*/*v*) containing 0.3% FA. All solutions were stored protected from light in amber glass vials certified for mass spectrometry from Waters (Milford, MA, USA).

### 3.2. LC-MS/MS Instrumentation and Conditions

LC-MS/MS analysis was performed on a Waters Alliance e2695 chromatographic system with an autosampler coupled to a Waters Micromass Quattro Micro triple-quadrupole mass spectrometer equipped with an electrospray ion source (ESI). Data processing was performed using Waters MassLynx 4.1 software. Separations were obtained on a reverse-phase Hypersil Gold PFP (50 × 2.1 mm; 5.0 µm) from Thermo Fisher Scientific (Waltham, MA, USA), maintained at room temperature and equipped with a guard column (PFP, 10 × 2.1 mm). The mobile phase was a mixture of 0.3% aqueous FA (component A) and 0.3% FA in ACN (component B), at a constant rate of 0.25 mL/min and under the following composition gradient: 0–3.0 min, 2% component B, 3.0–4.5 min, linear gradient from 0% to 50% component B, 4.5–7.5 min, 50% component B, 7.5–9.0 min, from 50% to 95% component B, 9.0–12.0 min at 95%, 12.0–13.5 min from 95% to 2% component B, and 13.5–16.0 min at 2%. The total run time was 16 min, including column re-equilibration. The injection volume was 5 μL.

Multiple reaction monitoring (MRM) transitions were used, acquired in both positive and negative ionization modes (ESI+, ESI−) and exploiting two different exclusive transitions for each analyte: The most abundant one for quantitative purposes and the second most abundant for identity confirmation. The optimised parameters were as follows: Ion source voltage, 4.3 kV; ion source temperature, 140 °C; desolvation temperature, 300 °C; desolvation gas flow, 550 L/h (nitrogen as the desolvation gas, argon as the collision gas); dwell time, 300 ms for all compounds. The precursor ions and the product ions, and collision energy were optimised and are shown in Table 1.

### 3.3. Microsampling: VAMS Collection and Pretreatment

Mitra^®^ VAMS microsamplers (10 µL) were provided by Neoteryx (Torrance, CA, USA) and include a polypropylene handle (approximately 4 cm long) topped with a small tip (approximately 2 mm in diameter) of a proprietary polymeric porous material (Figure 2a).

Whole blood VAMS microsamples (10 μL) were collected by putting the tip of a VAMS device in contact with the blood surface at a 45° angle for 3 s for complete tip saturation. The device was dried at RT for 1 h and stored at RT in the dark for 1 month at most, in a dedicated clamshell in a sealed polyethylene bag containing desiccant. To obtain fortified samples, after matrix drying on the VAMS device, methanolic working solutions (10 μL) containing the analytes and/or the ISs at a known concentration were added to VAMS tips and left to dry for 30 additional minutes before extraction.

VAMS tips, once detached from the plastic handler, were extracted with 100 µL of an ACN/H_2_O, 65:35, *v*/*v* mixture. The sample was subjected to UAE at 40 kHz for 10 min and VAE for 10 min. The samples were then centrifuged for 5 min at 4500× *g*, and the obtained supernatant was directly injected into the LC-MS/MS system.

### 3.4. Method Qualification

The VAMS-LC-MS/MS method was assessed in order to fulfil the European Medicines Agency (EMA) guidelines [35]. The tested parameters were linearity (including LOQ), absolute recovery, precision, matrix effect, stability, and accuracy.

Calibration standards as well as QC samples were prepared by fortifying whole blood VAMS samples from mice not subjected to any treatment for the assessment of linearity, sensitivity, inter- and intra-day precision and stability. Dried microsamples were spiked with 10 μL of working solutions containing the analytes at seven different concentrations and the ISs at a constant concentration, left to dry, then subjected to sample pretreatment and analysed by LC-MS/MS. The analysis was carried out in triplicate for each concentration.

The obtained analyte/IS peak area ratios were plotted as a function of the nominal added concentrations (ng/mL), and the least-squares method was used to obtain calibration curves. A 1/x^2^ weighing factor was applied. LOQ was calculated as the lowest concentration of analyte that could be quantified reliably, with acceptable accuracy (± 20%) and precision (%RSD < 20%).

Absolute recovery was evaluated on blood VAMS samples fortified with analyte standard solutions at three different concentrations (corresponding to low, intermediate and high calibration points for each analyte) and subjected to the previously described VAMS extraction procedure. The obtained analyte peak areas were compared with those obtained by analysing extracts from VAMS samples fortified post-extraction with the same nominal concentrations, and absolute recoveries were expressed as percentage. The acceptability criterion was absolute recovery > 80%. Precision was determined on the same fortified VAMS samples: Six replicates were analysed on the same day to assess intraday precision and over six different days to assess interday precision, expressed as RSD%. The acceptability criteria were RSD < 10% for intraday precision (<15% for the LOQ) and RSD < 15% for interday precision (<20% for the LOQ).

The IS-corrected matrix effect was evaluated by analysing six VAMS replicates, fortified post-extraction by adding known analyte concentrations at the same levels as precision assays. The mean analyte/IS peak area ratios for each added concentration was compared with analyte/IS peak area ratios from standard solutions at the same theoretical concentration and the resulting percentage was calculated. Acceptability criterion was a response in the 85–115% range.

Carryover was assessed by the injection of a blank solvent (50:50 water/ACN mixture containing 0.3% FA) after the highest concentration of the calibration curves (*n*  =  3). Carryover was considered acceptable if the signal at the retention time of the target analyte was less than 20% of the LLOQ value.

To test mid-term analyte stability, blood VAMS samples were stored at RT in the dedicated clamshell, protected from light, in sealed plastic bags containing desiccant for a time period of one month. At regular intervals (1 week), samples were pretreated and analysed (*n* = 3). The measured analyte concentrations were compared to those from samples extracted and analysed immediately after sampling and drying (t_0_) to assess the percentage of analyte loss. For autosampler and benchtop stability of processed VAMS, samples were pretreated and the extracts were stored for 24 h in the autosampler at RT and on the benchtop exposed to normal laboratory conditions, respectively, before re-analysis. Samples were considered stable when the bias from nominal concentrations was within ±15%.

Recovery assays were carried out in order to evaluate method accuracy: 10 μL of working standard mixtures at low, intermediate and high concentrations, and fixed amounts of the ISs, were added to dried blood VAMS sample replicates whose analyte concentrations were already assessed, and the samples were dried and analysed. Accuracy, expressed as percent recovery, was calculated by comparing the concentrations obtained from the fortified samples with the concentration in non-fortified samples plus the nominal concentration of the added standard mixture, and the recovery was considered acceptable if >80%.

## 4. Conclusions

In this study, an LC-MS/MS method was developed and optimised for the analysis of TRP and related biomarkers pertaining to the KYN pathway, the IND and 5-HT pathway or the gut TRP metabolism. Twenty-four target biomarkers were determined, for the first time, in dried whole blood microsamples after collection with VAMS samplers. The extraction of such biomarkers was optimised and finally carried out with 100 μL of an ACN/H_2_O mixture, exploiting a combination of UAE and VAE. Satisfactory recovery values were obtained for all analytes after this feasible and straightforward extraction procedure (>85%). The method exhibited good selectivity, correlation coefficient values for the calibration curves were >0.9987 for all target compounds and LOQ values ranged from 0.1 to 25 ng/mL. The intra- and inter-day precision values in terms of RSD were <9.6%. Moreover, the developed method was successfully applied for the analysis of biological samples from mice, and all compounds were detectable in whole blood microvolumes as low as 10 μL. In addition, no stability issues were observed when drying and storing VAMS samples at room temperature, as all tested compounds were stable for at least 30 days (analyte losses < 14%). Such results demonstrate great possibilities for VAMS sampling and analysis in biomarker and targeted metabolomics studies, and specifically for the assessment of TRP-related compounds to be applied in different research frameworks.

## Figures and Tables

**Table 1:** Multiple reaction monitoring (MRM) transitions and compound-specific MS parameters.

Analyte	MW (g/mol)	Q1 (*m*/*z*)	Q3 (*m*/*z*)	Collision Energy (eV)
TRP	204.22	205.24	188.0	15
NFK	236.22	237.24	142.2	10
KYN	208.21	209.30	192.0	10
AA	137.14	138.16	120.0	10
KYNA	189.17	190.20	144.0	20
3-HKYN	224.21	225.19	208.1	10
XA	205.17	206.21	160.1	15
3-HAA	153.14	154.20	136.1	15
QUIN	167.12	168.14	150.1	10
PIC	123.11	122.09	78.1	15
2-AMA	157.12	156.15	66.0	15
5-HTP	220.22	221.21	204.0	10
5-HT	176.21	177.22	160.1	10
5-HIAA	191.18	192.21	160.2	15
MEL	232.28	233.31	173.9	15
IND	117.15	118.09	91.1	20
TA	160.22	161.27	144.1	10
IDE	161.20	162.17	144.1	10
IPA	189.21	190.23	130.2	10
ILA	205.21	204.22	158.1	15
IAA	175.18	176.18	130.2	15
SK	131.17	132.20	117.1	20
IALD	145.16	146.18	118.1	15
IA	187.19	188.19	115.1	10
IS1	209.26	210.31	192.0	15
IS2	194.20	195.23	149.1	20
IS3	180.24	181.22	160.1	10
IS4	124.19	125.09	98.2	20

**Table 2:** Absolute recovery, precision and matrix effect data obtained on fortified whole blood VAMS samples.

Compound	Concentration Level ^a^	Intraday Precision (%RSD) ^b^	Interday Precision (%RSD) ^b^	Absolute Recovery (%) ^c^	Matrix Effect (%) ^c^
TRP	Low	7.9	9.5	86	88
	Medium	6.8	8.3	88	90
	High	6.3	7.8	93	92
NFK	Low	6.6	7.4	91	88
	Medium	6.2	6.8	93	92
	High	5.7	6.2	96	93
KYN	Low	7.7	8.9	88	89
	Medium	6.6	7.8	91	93
	High	6.1	7.3	94	93
AA	Low	6.3	7.2	88	87
	Medium	6.0	6.5	92	90
	High	5.5	5.9	95	94
KYNA	Low	6.4	7.1	87	89
	Medium	5.9	6.6	92	93
	High	5.6	5.8	93	95
3-HKYN	Low	6.0	7.0	90	90
	Medium	5.8	6.2	94	93
	High	5.3	5.7	94	96
XA	Low	6.3	6.8	87	91
	Medium	5.9	6.3	89	92
	High	5.2	5.6	93	95
3-HAA	Low	6.0	6.8	88	91
	Medium	5.6	6.1	91	91
	High	5.3	5.5	95	93
QUIN	Low	6.4	7.4	90	89
	Medium	5.8	6.7	91	94
	High	5.2	6.1	94	94
PIC	Low	6.7	7.6	89	92
	Medium	5.9	7.0	93	93
	High	5.7	6.3	94	94
2-AMA	Low	5.5	6.3	88	90
	Medium	5.1	5.6	92	93
	High	4.8	5.0	96	95
5-HTP	Low	5.6	6.2	89	92
	Medium	5.2	5.5	93	95
	High	4.8	4.9	94	96
5-HT	Low	6.9	7.7	90	88
	Medium	6.5	7.1	95	91
	High	6.0	6.5	96	92
5-HIAA	Low	5.8	6.1	88	90
	Medium	5.1	5.7	89	94
	High	4.9	4.8	92	94
MEL	Low	6.1	6.5	90	92
	Medium	5.5	6.0	90	95
	High	5.1	5.6	93	95
IND	Low	5.7	6.3	87	91
	Medium	5.3	5.6	88	92
	High	4.9	5.0	89	94
TA	Low	5.6	6.4	89	92
	Medium	5.3	5.6	92	95
	High	5.0	4.9	92	96
IDE	Low	5.5	6.3	88	93
	Medium	5.3	5.4	90	93
	High	4.8	4.9	92	95
IPA	Low	6.8	7.4	89	91
	Medium	6.3	6.8	91	94
	High	5.8	6.2	93	96
ILA	Low	6.6	7.1	90	88
	Medium	6.2	6.5	92	91
	High	5.5	6.0	94	95
IAA	Low	6.8	7.4	89	89
	Medium	6.3	6.8	93	92
	High	5.8	6.2	95	96
SK	Low	5.3	6.0	87	90
	Medium	5.0	5.2	90	93
	High	4.5	4.7	92	96
IALD	Low	6.0	6.5	90	92
	Medium	5.5	6.0	90	92
	High	5.0	5.6	93	94
IA	Low	6.0	6.8	90	90
	Medium	5.6	6.1	93	93
	High	5.3	5.5	96	96
IS1	\	3.2	3.9	95	93
IS2	\	3.5	4.0	97	95
IS3	\	3.8	4.2	93	98
IS4	\	3.3	3.9	93	96

^a^ In reference to each respective calibration curve: “Low” = close to the LOQ; “High” = close to the upper linearity limit (ULOQ); “Medium” = average of LOQ and ULOQ. ^b^ *n* = 6. ^c^ *n* = 3.

**Table 3:** Linearity and sensitivity data.

Analyte	Linearity Range (ng/mL)	r^2^	LOQ (ng/mL)
TRP	25–10,000	0.9988	25
NFK	0.3–100	0.9991	0.3
KYN	5–1000	0.9989	5
AA	0.2–100	0.9993	0.2
KYNA	0.2–100	0.9992	0.2
3-HKYN	0.2–100	0.9993	0.2
XA	0.2–100	0.9994	0.2
3-HAA	0.2–100	0.9992	0.2
QUIN	1–500	0.9991	1
PIC	1–500	0.9990	1
2-AMA	0.1–50	0.9995	0.1
5-HTP	0.1–50	0.9994	0.1
5-HT	1–500	0.9991	1
5-HIAA	0.1–50	0.9996	0.1
MEL	0.2–100	0.9994	0.2
IND	0.1–50	0.9994	0.1
TA	0.1–50	0.9992	0.1
IDE	0.1–50	0.9993	0.1
IPA	1–500	0.9989	1
ILA	1–500	0.9991	1
IAA	1–500	0.9991	1
SK	0.1–50	0.9994	0.1
IALD	0.2–100	0.9992	0.2
IA	0.2–100	0.9993	0.2

**Table 4:** Results from VAMS-LC-MS/MS analysis on real samples.

Individual	1	2	3
Sex	F	M	F
Blood VAMS Concentrations (ng/mL)			
TRP	6544	7620	7481
NFK	17.3	17.2	17.0
KYN	359	336	342
AA	8.2	7.5	7.4
KYNA	9.4	9.0	8.6
3-HKYN	9.0	9.7	12.2
XA	7.4	7.7	7.0
3-HAA	7.8	8.1	8.3
QUIN	49	52	55
PIC	55	51	51
2-AMA	5.0	5.5	6.1
5-HTP	1.4	3.2	2.3
5-HT	189	159	175
5-HIAA	3.1	2.7	2.7
MEL	15.0	12.9	13.2
IND	1.2	2.1	1.8
TA	1.3	1.5	1.7
IDE	0.3	0.3	0.3
IPA	166	168	187
ILA	226	230	255
IAA	300	311	296
SK	3.5	3.5	5.1
IALD	27.4	27.2	31.9
IA	11.6	11.6	16.5

## Data Availability

Data supporting reported results can be obtained from the authors.

## Sample Availability

Not available.
